# Feeding the world healthily: the challenge of measuring the effects of agriculture on health

**DOI:** 10.1098/rstb.2010.0122

**Published:** 2010-09-27

**Authors:** Sophie Hawkesworth, Alan D. Dangour, Deborah Johnston, Karen Lock, Nigel Poole, Jonathan Rushton, Ricardo Uauy, Jeff Waage

**Affiliations:** 1 Nutrition and Public Health Intervention Research Unit, London School of Hygiene and Tropical Medicine, Keppel Street, London WC1E 7HT, UK; 2 Leverhulme Centre for Intergrative Research in Agriculture and Health London International Development Centre, 36 Gordon Square, London WC1H 0PD, UK; 3 School of Oriental and African Studies, University of London, Thornhaugh Street, Russell Square, London, WC1H 0XG London, UK; 4 Royal Veterinary College, Hawkshead Lane, North Mymms, Hatfield, Hertfordshire AL9 7TA, UK; 5 London International Development Centre, 36 Gordon Square, London WC1H 0PD, UK

**Keywords:** health, nutrition, agriculture, food consumption, food policy

## Abstract

Agricultural production, food systems and population health are intimately linked. While there is a strong evidence base to inform our knowledge of what constitutes a healthy human diet, we know little about actual food production or consumption in many populations and how developments in the food and agricultural system will affect dietary intake patterns and health. The paucity of information on food production and consumption is arguably most acute in low- and middle-income countries, where it is most urgently needed to monitor levels of under-nutrition, the health impacts of rapid dietary transition and the increasing ‘double burden’ of nutrition-related disease. Food availability statistics based on food commodity production data are currently widely used as a proxy measure of national-level food consumption, but using data from the UK and Mexico we highlight the potential pitfalls of this approach. Despite limited resources for data collection, better systems of measurement are possible. Important drivers to improve collection systems may include efforts to meet international development goals and partnership with the private sector. A clearer understanding of the links between the agriculture and food system and population health will ensure that health becomes a critical driver of agricultural change.

## 1. Introduction

The relationship between agricultural production and population health is complex. Patterns of production lead to patterns of availability, price and distribution of food commodities. These raw ingredients are then processed in increasingly complex ways by the food manufacturing system and the combined effects of food production and processing influence individual food consumption and thereby population health. Besides these primarily nutritional links, agricultural and food systems act as conduits of food-borne and zoonotic disease and agrochemical pollutants and compete with the water supply and sanitation needs of local communities. In the context of international development, the interaction between health, agricultural productivity and income is particularly important since more than half of the world's poorest people live in farming communities, including many suffering from under-nutrition. Finally, the various interactions between agriculture, food and health increasingly play out on a global stage, with food produced in one region frequently consumed in another, mediated by trade liberalization and growing multinational food production and distribution industries.

To a large extent, global food production has kept up with the demands of a growing human population (Dyson 1996), but inequalities remain in regional and national distribution of the available food (Sen 1981). Recent estimates suggest that globally the combined effect of inadequate macro (protein–energy)- and micro-nutrient (including iron and iodine) intakes underpin 35 per cent of all child deaths and are responsible for 11 per cent of the global disease burden (Black *et al*. 2008). At the other extreme, excess dietary consumption, or over-nutrition, is increasingly leading to global epidemics of obesity and diabetes resulting in rapidly increasing burdens of disability and death affecting all world regions (WHO/FAO 2003; Haslam & James 2005). Indeed, several nutrition-related chronic diseases such as coronary heart disease and stroke are now among the leading causes of death worldwide, with the burden growing most rapidly in the world's lowest income countries (WHO 2008), often leading to a ‘double burden’ of both under- and over-nutrition, placing a huge burden on societies and the existing health systems (FAO 2006).

There remains a clear challenge to define ways in which agricultural production could better contribute, through the food chain, to improved health for all people. To achieve this, we need to understand the interactions between agriculture, food systems and health and to have tools that allow us to predict the effects on health of agricultural change and innovation. In this paper, we explore our capacity to measure and predict agricultural impacts on health, focusing particularly on nutrition. We begin by pulling together the diverse current literature on nutrition and health to identify what constitutes a healthy diet. We then examine how we currently measure food availability and consumption in different populations, looking particularly at our capacity to do this on a global scale. Finally, we explore whether, given the tools currently at our disposal, we are able accurately to assess the impact of changes in agriculture and food systems on population health and the potential for health to act as a driver to stimulate these changes.

## 2. The components of a healthy diet

It has long been recognized that a balance of nutrients forms the basis of a healthy diet, and ongoing research continues to further our understanding in this area. The primary elements of a diet are the three macronutrients, carbohydrates, protein and fat (table 1), but the relative contribution of these macronutrients and their constituent sub-types to the diet are crucially important in the definition of a healthy diet.

**Table 1.** The components of a healthy diet and population nutrient intake goals from the WHO Expert Committee. Source: WHO/FAO (2003).

component	dietary sources	recommendations^a^
carbohydrate	staple crops such as rice, wheat and potatoes as well as simple sugars (see below)	55–75%
free sugars^b^	added sugar (often fructose and sucrose) plus naturally occurring sources such as honey and fruit juices	<10%
fat^c^		15–30%
saturated fatty acids	animal sources including meat and butter as well as vegetable sources including coconut and palm oil	<10%
*n*−6 polyunsaturated fatty acids^d^	most abundant in seed oils such as corn and sunflower fatty acids	5–8%
*n*−3 polyunsaturated fatty acids^e^	found in canola and soya oil as well as oily fish	1–2%
*trans* fatty acids	produced^f^ during industrial manufacture of partially hydrogenated vegetable oils and found in many fried and baked goods	<1%
monounsaturated fatty acids	preponderant in some oils such as olive oil	by difference^g^
protein	animal products including meat and milk, vegetable sources including legumes	10–15%
sodium chloride	salt	<5 g d^−1^
fruits and vegetables	fruits are the seed-containing part of the plant while vegetables in this context are the remaining edible parts	≥400 g d^−1^
total dietary fibre	whole-grain cereals, fruits and vegetables	from foods

^a^Recommendations refer to population nutrient intake goals defined by the WHO Expert Committee (WHO/FAO 2003).

^b^Free sugars refers to all ‘simple’ sugars (monosaccharides and disaccharides) added to foods by the manufacturer, cook or consumer, as well as naturally occurring sugars.

^c^Fats are categorized by the absence (saturated) or presence (unsaturated) of double bonds, the number of double bonds (one, monounsaturated; more than one, polyunsaturated) and their position in the carbon chain.

^d^*n*−6 indicates that the first double bond occurs on the sixth carbon in the fatty acid chain while polyunsaturated indicates the presence of more than one double bond.

^e^*n*−3 indicates that first double bond occurs on the third carbon in the fatty acid chain while polyunsaturated indicates the presence of more than one double bond.

^f^A very small proportion of TFAs in the diet are naturally occurring and are found in foods from ruminant animals.

^g^Monounsaturated fat = total fat−(saturated fat + polyunsaturated fat + *trans*-fat).

### (a) Carbohydrates

Carbohydrates are the predominant source of energy in the diet, playing a key role in metabolism and the maintenance of homeostasis. The type and balance of carbohydrates in the diet are of great importance to health. For example, the consumption of foods containing large amounts of simple carbohydrates (refined sugars), such as sweetened beverages, can promote weight gain by increasing the energy density of the diet and by their lower satiety value (van Dam & Seidell 2007). While, in contrast, diets rich in complex carbohydrates such as whole-grain cereals, vegetables and nuts contribute to lowering the risk of type 2 diabetes (de Munter *et al*. 2007; Barclay *et al*. 2008), cardiovascular disease (Streppel *et al*. 2005) and certain types of cancers (World Cancer Research Fund/American Institute for Cancer Research 2007), while also providing a good source of fibre and a range of vitamins and minerals. The most recent FAO/WHO Scientific Update on carbohydrates in human nutrition stated that ‘whole-grain cereals, vegetables, legumes and fruits are the most appropriate sources of dietary carbohydrate’ (Mann *et al*. 2007).

### (b) Fats

Fats are a second major dietary energy source and are essential for growth and development in early life. The fat in our diets is composed mainly of fatty acids, which vary widely in their carbon chain length and the number and position of their double bonds (table 1). It is increasingly recognized that different structural categories of fats have contrasting impacts on health (Lecerf 2009). For example, there is strong evidence that the consumption of *trans*-fatty acids (TFAs) increases the risk of cardiovascular disease, with potential adverse effects also on insulin resistance and adiposity (Teegala *et al*. 2009). In contrast, the omega-3 long-chain polyunsaturated fatty acids (omega-3 LCPs), most commonly found in fish, have been shown to have beneficial effects for cardiovascular health (Scientific Advisory Committee on Nutrition 2004; Lecerf 2009). Omega-3 LCPs play a crucial role in brain and retinal development *in utero* (Uauy & Dangour 2006), but evidence is inconsistent that additional consumption of these oils in childhood enhances brain function. There is also no evidence that consuming supplemental omega-3 LCPs in later life helps slow cognitive decline (Dangour *et al*. 2010).

### (c) Protein

Dietary intake of protein is vital for normal growth and development and the maintenance of body protein (WHO/FAO/UNU 2007). Proteins are composed of amino acids, some of which cannot be synthesized in the body and thus are termed ‘essential’, and the quality of protein in a diet is defined based on its provision of essential amino acids. The digestibility of proteins is also an important factor in defining dietary protein adequacy, with protein sources in typical Western diets having a digestibility of approximately 95 per cent, while proteins from a typical Indian rice-based diet have a digestibility of only 77 per cent (WHO/FAO/UNU 2007). Beyond the primarily metabolic demand, attention is now focusing on the role of protein intakes in promoting lifelong health and there is emerging evidence that protein quality may have consequences for optimal muscle and bone growth (Millward *et al*. 2008). The most recent expert consultation on protein requirements stated that an intake of 0.83 g of high-quality protein per kilogram of body weight per day should be sufficient to meet the requirements of most of the adult population and highlighted that intakes three to four times higher than this may not be risk free (WHO/FAO/UNU 2007).

### (d) Nutrients as foods

In reality, diets are not categorized based purely on their macronutrients content, but instead are composed of different foods providing specific combinations of macro- and micro-nutrients. One of the most diverse food groups is fruits and vegetables, which play an important role in promoting health. No single known component nutrient explains the observed beneficial health effects of consuming a high vegetable and fruit diet and their impact is likely due to a combination of being low in energy density, high in fibre and a source of vitamins and minerals as well as to lesser-understood bioactive components such as polyphenols. The protective effect of fruit and vegetable consumption on cardiovascular disease and other chronic disease risk is well recognized (WHO/FAO 2003), and it has been estimated that 2.6 million deaths per year could be attributed to the inadequate consumption of fruit and vegetables, primarily through their effects on ischaemic heart disease and stroke (Lock *et al*. 2005).

In some countries and cultures, meat and dairy products are an important part of the diet, representing good sources of protein and a range of minerals such as iron, zinc and calcium and micro-nutrients such as vitamin B12. In contexts where dietary intakes are sub-optimal, animal source food products can be an essential source of these important nutrients. However, some meat and dairy products are also a major contributor of saturated fat in the human diet, and high intake of saturated fat is consistently associated with increased risk of heart disease, largely because of the effect on serum cholesterol concentrations (Hu *et al*. 2001; WHO/FAO 2003; Jakobsen *et al*. 2009). High consumption of red (and processed) meat has also been shown to be associated with increased risk of colorectal cancer (World Cancer Research Fund/American Institute for Cancer Research 2007) and total mortality (Sinha *et al*. 2009).

### (e) Synthesis of expert reports on dietary intake for the prevention of disease

There are evident complexities in defining the relationships between population nutritional intake and health. It is therefore a challenge to provide comprehensive dietary guidelines for population intakes based on the global diversity of primary foodstuffs. Dietary guidelines have been part of public health nutrition policies since the early twentieth century. These guidelines, often produced by expert bodies, initially focused on the prevention of specific nutrient inadequacies, but more recently, their focus has changed to the prevention of food and nutrition-related chronic diseases. However, expert reports rarely synthesize evidence into dietary guidelines that encompass nutritional inadequacy, infectious and chronic disease.

This shortcoming was recently addressed in a systematic review of expert panel dietary recommendations for the prevention of nutritional deficiencies, infectious and chronic diseases published between 1990 and 2004 (World Cancer Research Fund/American Institute for Cancer Research 2007). The review identified 94 expert reports of which only three (two from India and one from South Africa) arose from expert panels in low-income countries. The reviewers identified a broad consensus in dietary recommendations for the prevention of disease (table 2). Generally, reports recommended diets high in cereals, vegetables, fruits and pulses and low in red and processed meats. Recommended diets are correspondingly high in dietary fibre and micro-nutrients and low in fats, saturated fatty acids, added sugars and salt (World Cancer Research Fund/American Institute for Cancer Research 2007).

**Table 2.** Summary of expert panel dietary recommendations for the prevention of nutritional deficiencies, infectious and chronic diseases. Source: adapted with permission from World Cancer Research Fund/American Institute for Cancer Research (2007). CHD, coronary heart disease; CVD, cardiovascular disease; LDL, low density lipoprotein.

exposure	recommendations^a^	to prevent or manage
cereals (grains), roots, tubers and plantains	include whole-grain cereals in the diet with a suggested intake of three to six or more servings per day	CVD, CHD
	foods high in iron should be eaten in combination with foods that enhance rather than inhibit iron absorption: cereals (grains) should be consumed with meals of low iron content, and foods high in ascorbic acid, such as tubers, should be included with meals.	iron deficiency anaemia
vegetables, fruits, pulses (legumes), nuts, seeds, herbs and spices	include 400 g (five or more servings) per day of vegetables and fruits, including pulses (legumes)	CVD, CHD, hypertension
	foods high in ascorbic acid, such as orange juice, carrots and cauliflower, should be included with meals	iron deficiency anaemia
meat, fish, and eggs	red meat consumption should be moderated and lean meat preferred (unspecified amount)	CVD, CHD
	consume between one to three servings per week of fish, choosing oily fish	CVD, CHD
fats and oils	limit intake of hydrogenated/partially hydrogenated vegetable oils and hard margarines (unspecified amount)	CHD
	total dietary fat to provide no more than 30–35% of total energy (intake should not be restricted in children under 2 years)	CVD, CHD, overweight/obesity
	intake of saturated fat should be no more than 7–10% of energy	CVD, CHD
	restrict intake of myristic acid (including coconut products)	CHD
	limit intake of dietary cholesterol to <300 mg d^−1^ (intake less than 200 mg d^−1^ for individuals at risk or with pre-existing CVD)	CVD, CHD
	limit intake of polyunsaturated fatty acids to no more than 10% of energy	CHD
	limit intake of TFAs (unspecified amount)	CVD
salt and sugar	limit intake of sodium to no more than 100 mmol d^−1^	hypertension
	limit/reduce consumption of salt and salted foods to no more than 6 g of salt d^−1^	CVD, CHD, hypertension, stroke
	limit the proportion of energy in the diet from sugar (unspecified amount)	CVD, overweight/obesity, dental caries
	avoid consumption of sugary foods and drinks between meals	dental caries
milk and dairy products	eat low-fat versions of dairy products in preference to high-fat versions	CVD, CHD
water, fruit juices, soft drinks, and hot drinks	avoid using sugary drinks in baby bottles	dental disease
alcoholic drinks	limit intake of alcoholic drinks to two drinks for men and one drink for women per day and if drinking, do so only with meals	CVD, CHD, hypertension, stroke
food production, processing, preservation and preparation	limit/reduce intake of refined carbohydrates/grain products and foods	CVD
dietary constituents and supplements	include fibre in the diet (unspecified amount)	CVD
	ensure an adequate intake of vitamin D and calcium	osteoporosis

^a^Synthesis of recommendations from a systematic review of expert reports published since 1991. Recommendations have only been included if they were made in three or more reports.

In 2003, WHO published population nutrient intake goals (WHO/FAO 2003) which continue to reflect the current evidence and provide a simple definition of the nutritional composition of a ‘healthy diet’ for nine billion people (table 1). The WHO report did not focus on the micro-nutrient intake requirements, although this continues to be an active area of research (FAO/WHO 2002). Currently, the WHO recommends, among others, vitamin A supplementation to children in at-risk areas (de Benoist *et al*. 2001), salt iodization to prevent iodine-deficiency disorders (WHO 1994) and either iron fortification or supplementation for the prevention of iron deficiency anaemia (WHO/UNICEF/UNU 2001).

## 3. Food consumption trends

Evidence from around the world suggests that economic development results in major transitions in population-level dietary, and corresponding disease, patterns. The nutrition-related changes (encompassing both dietary intake and physical activity) have been termed the ‘nutrition transition’ and describe trends moving away from dietary patterns that typify those of hunter–gatherers containing large amounts of fibre and low amounts of sugar and fat to energy-dense diets composed predominantly of highly processed foodstuffs common to much of the developed world today (Drewnowski & Popkin 1997; Popkin 2004, 2006). The dietary changes are themselves driven by a variety of culturally specific factors including the increased production, availability and marketing of processed foods and the complex effects of urbanization (Popkin 2006).

The future prospects look bleak as societal change in low- and middle-income countries is accelerating the nutrition transition (Popkin 2002). Furthermore, as rural to urban migration continues, there will be increasing dependency on complex food chains, which implies that the direction of these dietary transitions is and will be one way. The consequences for population prevalence of nutrition-related chronic disease are all too evident; the WHO Global Burden of Disease project lists coronary heart disease and stroke within the top 10 leading causes of death worldwide with diabetes mellitus also a leading cause of death in high- and middle- and increasingly in low-income countries (WHO 2008).

## 4. Assessing global food availability and individual food consumption

Changing patterns of agricultural production, food availability and processing will have profound impacts on individual food consumption and, as a result, on population health. A thorough understanding of these impacts requires a dependable means of measuring food consumption around the world. In the following sections, we compare the methods currently used to assess food consumption, particularly the estimation of food consumption from patterns of food production and availability through food balance sheets (FBS), from studies of food purchases as part of household budget surveys (HBS) and from individual dietary surveys. These methods are also critiqued elsewhere in this supplement as part of an analysis of food consumption trends (Kearney 2010).

### (a) Commodities production, FBS and global food availability

The United Nations Food and Agriculture Organization (FAO) compiles national data on food production and on *per capita* food availability for most countries in the world. These data are available online (http://faostat.fao.org) and are widely used to inform agricultural and food policy. Production data are presented for the top 20 most important food and agricultural commodities produced in a given country in terms of their value and size. Food availability data are presented in FBS and provide figures on the estimated availability of over 100 foodstuffs in grams per capita per day. The FBS are constructed using FAO information on food production and net trade. The food available for consumption is then calculated after estimating the amount used for industrial or agricultural purposes (for example, as seed or for animal consumption or bio-fuels), wastage in the production system and change in national stock levels. It is important to emphasize that measures of food availability are not measures of food consumption, but in the absence of other data, food availability is widely used as a proxy for food consumption.

The calculation of food availability is subject to a range of potential errors, from the initial calculation of production and trade to the determination from this of what food is available for consumption. The statistics used for food production and net food trade by FAO have been criticized by both academics (Svedberg 1999) and independent evaluators (CC-IEE 2008). In 2008, an independent evaluation noted that ‘the quantity and quality of data coming from national official sources has been on a steady decline since the early 1980s’ (CC-IEE 2008). This lack of good quality data is particularly acute for certain developing countries where there may be no official statistics; FAO currently fills this gap by providing its own modelled or imputed estimates of food production, which are used for over 70 per cent of African countries and for over 50 per cent of countries from Asia and the Pacific (CC-IEE 2008).

Figures on animal populations and production parameters provide further illustration of errors inherent in office-based estimates. A recent case study from South America revealed that livestock population figures reported by the FAO differed by 10–50% from the reality on the ground and that very sparse data on livestock production parameters were used to estimate production (Rushton & Viscarra 2010). A striking example from this region is the difference in estimates in Brazilian cattle populations, with the official number being 180 million compared with estimates of 160 million (FNP Consultoria e Comercio 2006). As agricultural production numbers form the basis of FBS estimates of food availability, errors of this magnitude will have important consequences for the accuracy of the resulting food availability data, and any estimates of consumption calculated from these.

At the level of estimating *per capita* food availability, errors in FBS estimates can result from incomplete or out-of-date country-specific population estimates which are usually based on the resident population and do not take into account tourists, illegal immigrants or refugees. This issue may be particularly pronounced for many sub-Saharan African countries where published population census data are often out of date and are likely to suffer from undercounting and misreporting due to issues of accessibility, risk and the conceptual problems of encompassing highly mobile populations and complex patterns of household formation (Sender *et al*. 2005).

FBS data provide incomplete information on the level of home production of foods or on the level of processing different food commodities undergo prior to their availability for consumption. In many low-income countries, foods produced at home (which do not reach the market) remain largely unprocessed and are predominant in the household diet. In contrast, as countries undergo the nutrition transition, foods are often highly processed, and FBS data based on the production and trade of agricultural commodities are unable to provide information on the composition of the processed foods actually available for consumption.

Finally, a key source of error in using FBS food availability statistics as a proxy for food consumption is that FBS data do not allow for food waste at the retail and household level. This level of food wastage can be particularly high in urban areas of developed countries, but will vary greatly both between and within countries. In the UK, it has been estimated that one-third of all food purchases (i.e. foods available for consumption at the household level) are thrown away, equating to 6.1 million tonnes of foodstuffs a year (WRAP 2008).

### (b) Food availability at the household level

HBS generally conducted by national statistical offices are available from many countries in the world including an increasing number of low-income countries (Smith *et al*. 2006). These surveys generally aim to acquire nationally representative information on household expenditure for a range of commodities, including food, primarily to construct cost-of-living indices. Where HBS include information on the quantities of different types of foods purchased, as well as consumption from own production, this information equates to the food available at the household level and is therefore frequently used as a proxy estimate of consumption in a manner similar to FBS food availability data. In HBS, dietary data are collected as part of the larger household level survey, which is a strength as they can be related to the socio-economic status of the household and, provided the sample is representative, regional variations can also be investigated. In reality, however, samples are not always representative due to issues such as a lack of accurate sampling frame, poor response rates and a tendency to over-sample urban compared with rural areas and poorer compared with wealthier households.

Other important limitations of using HBS data to assess the composition of the household diet include a lack of information on food consumed outside the home, on waste within the household or on food used for other reasons (such as pet food) or fed to guests. Measuring the consumption of home-produced food may also prove difficult. In addition, the methodologies used may not be directly comparable between countries (Naska *et al*. 2009). A further important limitation when using the data as a proxy for individual dietary intake is the lack of information on the distribution of food within the household. Intra-household food allocation may be a particular concern in low-income country settings where food consumption is known to vary widely between members of a household, with higher-status household members often consuming considerably more, and better quality, foods than other members of the family (Gomna & Rana 2007; Leroy *et al*. 2008). A final consideration is that seasonal trends in food consumption are not captured by these surveys unless they are conducted year-round, which has its own consequences in terms of implementation costs.

Few studies have quantitatively assessed the comparability of food availability data derived from FBS and HBS. However, a recent comparison of data from 18 European countries reported a general tendency for HBS-derived values to be lower than those from FBS for the major food groups (Naska *et al*. 2009). Despite the lower values in HBS, estimates from the two methods of the availability of most food groups, with the exception of meat products, correlated well (Naska *et al*. 2009). HBS and FBS are thus complementary methods of assessing food availability and have an important role to play in informing public policy. However, because of their inherent limitations, they are not able to provide accurate data on food consumption at the individual level (Serra-Majem *et al*. 2003); a concept that is explored further in the following sections.

### (c) Individual dietary surveys of food consumption

Direct estimates of individual food consumption for a population are generally derived from surveys conducted on nationally representative samples. When conducted properly, individual dietary intake data from population surveys can often be sub-divided by age and sex categories and used to investigate regional and socio-economic variations. There is a surprising paucity of nationally representative surveys even from high-income country settings. Indeed, in order to estimate the consumption of fruit and vegetables by individuals worldwide, the Global Burden of Disease project was only able to identify nationally representative dietary intake survey data from 26 countries and had to rely entirely on FBS food availability data for African countries (Lock *et al*. 2005). This lack of dietary intake surveys probably arises from the complexities and expense involved in conducting regular high-quality rounds of data collection and analysis, insufficient information on the energy and nutrient composition of local foods and low participant literacy levels in some countries (Ferro-Luzzi 2002).

Collecting individual dietary intake data involves methods such as weighed records, 24 h recalls and food frequency questionnaires, none of which is error free. Weighed food records over seven days are generally viewed as the ‘gold standard’ by nutritionists, although it is recognized that respondents must be highly motivated and literate and that the burden of data collection may impact on their dietary behaviour (Gibson 2005). Twenty-four-hour recall methods are commonly used, although must be repeated on several days to more accurately capture habitual dietary intake (Gibson 2005). Food frequency questionnaires require fewer resources, but there exists an ongoing debate around the validity of dietary intake data reported via this method (Bingham *et al*. 2003; Prentice 2003). Difficulties in comparison and interpretation of individual dietary intake data collected in different countries also arise from the use of diverse study designs, sampling frames, seasonal variation in dietary intake and methods of data collection.

### (d) A comparison of individual dietary intake data and FBS food availability data: UK and Mexico

In order to examine the challenges posed in the comparison of individual dietary intake surveys with the more globally available FBS data on food availability, we present an analysis involving national surveys of individual dietary intake and FBS food availability data from two countries: the UK and Mexico. We selected these two national surveys to compare countries at different stages of development from different regions of the world. We were greatly constrained by the need to find comparable dietary intake survey data, and in this regard, it is noteworthy that we found no low-income or lower middle-income countries for which national-level dietary intake survey data could be obtained.

The UK National Diet and Nutrition Survey (NDNS) recruited around 2000 adults individuals from across the UK and collected dietary information using a seven-day-weighed record (Henderson *et al*. 2003). The Mexican Health and Nutrition Survey (MHNS) included 20 000 adults and used a 101-item food frequency questionnaire to record foods eaten over the previous seven days (Ramirez *et al*. 2009). FBS food availability data from the same year that the surveys were conducted were extracted for both countries from the FAO website.

For both the UK and Mexico, individual dietary intake of all macronutrients was substantially lower than estimated to be available at a national level from FBS data (tables 3 and 4). In the UK and Mexico, energy availability was approximately 70 per cent and 83 per cent higher, respectively, than the average adult energy consumption as estimated from dietary intake surveys. These findings mirror those reported from a comparison of four other high-income countries (Canada, Finland, Poland and Spain), which also demonstrated that FBS food availability data overestimated actual food consumption (Serra-Majem *et al*. 2003). Similarly, FBS data on fruit and vegetable availability in 15 countries (mostly high income) have been reported to substantially over-estimate actual consumption, although the degree of overestimation varied widely (Pomerleau *et al*. 2003). It has been suggested that as the food system develops and becomes more complex, the discrepancy between dietary intake and food availability data increases due to a lack of information at the manufacturing level as well as the variations in waste (FAO 1983; Dowler & Ok Seo 1985; Sekula *et al*. 1991).

**Table 3.** Nutrient consumption of adults (19–64 years) in the UK.

	food available for consumption^a^	National Diet and Nutrition Survey^b^						healthy nutrient goals^c^
		male			female			
		all (*n* = 833)	benefits^d^ (*n* = 110)	no benefits (*n* = 723)	all (*n* = 891)	benefits^d^ (*n* = 150)	no benefits (*n* = 740)	
energy								
kcal per capita per day	3369	2321.6 (585.2)	2113.8 (597.1)	2355.0 (573.2)	1640.9 (420.4)	1521.4 (501.6)	1664.8 (406.0)	
% animal source	30							
carbohydrate								
gram per capita per day	425.3	275 (79)	259 (74)	277 (79)	203 (59)	193 (70)	205 (57)	
% of calories	50.5	47.7 (6.0)	48.4 (6.6)	47.6 (5.9)	48.5 (6.7)	49.7 (6.8)	48.3 (6.7)	55–75%
calories from free sugars^e^ (%)		13.6 (6.7)	14.5 (8.0)	13.5 (6.5)	11.9 (6.5)	13.6 (8.6)	11.5 (6.0)	<10%
fat								
gram per capita per day	141.3	86.5 (28.2)	81.5 (29.6)	87.2 (27.9)	61.4 (21.7)	56.4 (23.6)	62.5 (21.2)	
% of calories	37.7	35.8 (5.6)	35.8 (5.8)	35.8 (5.6)	34.9 (6.5)	34.4 (6.2)	35.0 (6.6)	15–35%
calories from saturated fat (%)		13.4 (2.9)	13.3 (3.2)	13.4 (2.9)	13.2 (3.3)	13.0 (2.8)	13.2 (3.4)	<10%
calories from *trans-*fat (%)		1.2^f^ (0.4)	1.2 (0.4)	1.2 (0.4)	1.2^f^ (0.4)	1.1 (0.4)	1.2 (0.4)	<1%
protein								
gram per capita per day	99.4	88.2 (32.7)	79.6 (26.0)	89.6 (33.4)	63.7 (16.6)	56.0 (18.5)	65.2 (15.8)	
% of calories	11.8	16.5 (3.6)	15.8 (3.5)	16.6 (3.6)	16.6 (3.5)	15.8 (3.5)	16.7 (3.5)	10–15%
fruit^g^								
gram per capita per day	232.3	260.1			205.0			
vegetables^h^								>400 g
gram per capita per day	228.8	230.4			255.6			

^a^Food balance sheet information, 2000: FAOSTAT (FAO 2009*a*).

^b^National Diet and Nutrition Survey, UK, 2000/2001 (Henderson *et al*. 2003). Values are means (s.d.) for intake derived from 7-day-weighed food record.

^c^Population nutrient intake goals defined by the WHO Expert Committee (WHO/FAO 2003).

^d^Benefits: refers to households in receipt of working families tax credit at the time of the interview of the receipt of income support or (income related) job seeker's allowance by the respondent or anyone in their household in the 14 days prior to the data of the interview.

^e^Free sugars defined as non-milk extrinsic sugars such as honey and table sugar.

^f^*Trans*-fat intake re-estimated as 1 per cent of food energy by the Food Standards Agency in 2007 to take into account the new product information.

^g^Fruit defined by FBS as: plantains, bananas, orange, lemons and limes, grapefruit and pomelos, tangerines, mandarins, clementines, satsumas, other citrus fruit, melons, watermelons, apples, apricots, avocados, cherries, figs, grapes, mangoes, papaya, peaches, pears, persimmons, pineapples, plums, quinces, blueberries, cranberries, gooseberries, raspberries, strawberries, kiwi, other fruits (fresh), dates, figs (dried), prunes, currants, raisins, other dried fruit. Fruit defined by NDNS as: apples and pears, citrus fruits, bananas, canned fruit in juice, canned fruit in syrup, ‘other fruit’ (including plums, grapes and soft fruits).

^h^Vegetables defined by FBS as: beets, carrots, turnips, rutabagas/swedes, onions (green), onions (dry), artichokes, tomatoes, asparagus, cabbage, cauliflower, celery, kale, lettuce, spinach, beans (green), broad bean (green), chilli peppers, garlic, cucumbers, mushrooms, eggplant, peas (green), pumpkins, squash, gourds, okra, radishes and other vegetables. Vegetables defined by NDNS as: raw carrots, raw tomatoes, ‘other raw’ and salad vegetables, peas, green beans, leafy green vegetables, carrots (not raw), tomatoes (not raw), baked beans, ‘other vegetables’ (including mushrooms, cauliflower, onions and peppers).

**Table 4.** Nutrient consumption of adults (20–59 years) in Mexico.

	food available for consumption^a^	Mexican Health and Nutrition Survey^b^				healthy nutrient goals^c^
		male (*n* = 5898)	female (*n* = 9848)	rural (*n* = 6466)	urban (*n* = 9280)	
energy						
kcal per capita per day	3244	1963 (1475, 2673)	1592 (1178, 2091)	1644 (1189, 2253)	1750 (1296, 2336)	
% animal source	19.8					
carbohydrate						
gram per capita per day	503.6	294.1 (218.1, 390.7)	243.2 (179.9, 324.1)	266.9 (194.7, 375.1)	260.5 (193.1, 350.2)	
% of calories	62.1	61.5 (55.5, 67.9)	61.5 (55.2, 67.8)	66.3 (59.9, 72.1)	60.6 (54.5, 66.4)	55–75%
fat						
gram per capita per day	96.0	55.0 (38.2, 77.4)	46.2 (30.9, 65.1)	40.2 (26.4, 59.2)	52.1 (36.2, 71.5)	
% of calories	26.6	26.4 (21.0, 31.4)	26.1 (20.9, 31.5)	22.1 (17.2, 27.8)	27.1 (22.2, 32.1)	15–30%
calories from saturated fat (%)		7.6 (5.4, 9.9)	7.6 (5.4, 10.2)	6.1 (4.1, 8.8)	7.8 (5.8, 10.3)	<10%
protein						
gram per capita per day	92.0	57.4 (42.8, 77.7)	49.2 (35.9, 65.1)	47.8 (34.8, 65.5)	53.8 (39.5, 70.5)	
% of calories	11.3	11.8 (10.5, 13.5)	12.0 (10.6, 13.7)	11.3 (10.3, 12.7)	12.1 (10.6, 13.8)	10–15%
fruit^d^						
gram per capita per day	316.4	52.3	75.5	68.9	64.6	>400 g
vegetables^e^						
gram per capita per day	167.4	50.2	61.2	53.6	58.1	

^a^Food balance sheet information, 2005: FAOSTAT (FAO 2009*a*).

^b^Mexican Health and Nutrition Survey (MHNS), 2005/2006 (Barquera *et al*. 2009). Data are median (inter-quartile range).

^c^Population nutrient intake goals defined by the WHO Expert Committee (WHO/FAO 2003).

^d^Fruit defined by FBS as: plantains, bananas, orange, lemons and limes, grapefruit and pomelos, tangerines, mandarins, clementines, satsumas, other citrus fruit, melons, watermelons, apples, apricots, avocados, cherries, figs, grapes, mangoes, papaya, peaches, pears, persimmons, pineapples, plums, quinces, blueberries, cranberries, gooseberries, raspberries, strawberries, kiwi, other fruits (fresh), dates, figs (dried), prunes, currants, raisins, other dried fruit. Fruit defined by MHNS as: fleshy edible parts from trees or fresh plants containing seeds.

^e^Vegetables defined by FBS as: beets, carrots, turnips, rutabagas/swedes, onions (green), onions (dry), artichokes, tomatoes, asparagus, cabbage, cauliflower, celery, kale, lettuce, spinach, beans (green), broad bean (green), chilli peppers, garlic, cucumbers, mushrooms, eggplant, peas (green), pumpkins, squash, gourds, okra, radishes and other vegetables. Vegetables defined by MHMS as: plants having edible parts such as leaves (cabbage, lettuce, spinach), stems (celery etc.), sprouts (asparagus etc.), flowers (cauliflower, artichoke etc.), pods (green beans, etc.), roots (carrots, beets etc.), blubs (onions, garlic, etc.), fruits culturally considered vegetables in Mexico (such as tomato, cucumber, avocado), green seeds (peas, broad beans) and pulses (beans, lentils, chickpeas and soya beans).

### (e) Using dietary intake surveys to assess population dietary adequacy

Population dietary intake data can be used to assess the adequacy of the diet, to highlight at-risk groups and to assess the effectiveness of interventions aimed at population dietary change. Data from the NDNS suggest that adults in the UK are on average exceeding the recommended intakes of free sugars, total fat and saturated fat (table 3). These average figures obscure what can be a wide variation in intakes; the range between the lowest and highest 2.5 percentile of percentage energy from fat for men was 24–47% and for women was 22–48% (Henderson *et al*. 2003). In contrast to the UK, total and saturated fat intakes in Mexico appear to lie within the range recommended as a healthy nutrient intake goal, although again these mean values obscure a range of intakes and some individuals will be consuming over 35 per cent of energy from fat. The intake of fats has been shown to increase as countries progress through the nutrition transition and this difference in intakes may reflect the different transition stages attained by the two countries (Popkin 2006). In Mexico, the fruit and vegetable intakes are much lower than the 400 g intake goal and may point to an area of health promotion that requires emphasis.

A significant shortcoming in the use and interpretation of FBS food availability data is that they provide no information on the variation of availability by sex, socio-economic status, region or age. Comprehensive national dietary intake surveys, such as the NDNS and MHMS, will stratify dietary intakes into sub-groups, thereby providing important insights into the differential burdens of disease risk factors in addition to highlighting at-risk groups.

For example, in the NDNS, low socio-economic status, defined as individuals receiving state benefits, was associated with greater intake of free sugars in both men and women (table 3). Such wealth-related differences in diet pattern are well recognized as one of the main causes of social inequalities in health (Robinson *et al*. 2004; Shelton 2005). Similarly, data from the MHNS showed that individuals from urban areas reported substantially higher intakes of fat and saturated fat than those in rural areas (table 4), highlighting one of the commonly observed trends associated with urbanization, which is in turn one of the key drivers of the nutrition transition (Drewnowski & Popkin 1997). From this brief synopsis of the nutritional intakes of two countries at different stages of development, we can see the wealth of information that may be derived from national surveys and the usefulness of this information for informing nutrition policy.

### (f) Using FBS estimates when dietary intake surveys are not available: nutrient availability in Bangladesh and Tanzania

Nationally representative nutritional surveys have not been conducted in the majority of low-income countries (Smith *et al*. 2006). In South Africa, for example, intake surveys have been carried out for particular regions or for particular population groups (children and pregnant women), but not for the population as a whole. In these settings, FBS food availability data are often used as a proxy for individual dietary intakes despite their important limitations outlined above. FBS data for Bangladesh and Tanzania suggest very low energy availability (table 5), which for Tanzania does not meet the World Food Programme target level of calorie consumption (2100 kcal d^−1^) (WFP 2007). In addition, only a small proportion of this energy is derived from animal sources, suggesting a diet that may be low in certain key vitamins and minerals that are less available from vegetable sources. The Bangladesh data also reveal a level of fat availability that is below the minimal desirable intake of 15 per cent of energy (FAO/WHO 1994). However, given the substantial limitations of using FBS food availability data as a proxy measurement of food consumption, it seems pertinent to question the validity of the data presented in table 5.

**Table 5.** Food availability information for Bangladesh and Tanzania.

	food available for consumption^a^		healthy nutrient goals^b^
	Bangladesh	Tanzania	
energy			
kcal per capita per day	2261	2019	
% animal source	3.3	6.9	
carbohydrate			
gram per capita per day	454.5	381.1	
% of calories	80.4	75.5	55–75%
fat			
gram per capita per day	27.6	33.5	
% of calories	11	14.9	15–30%
protein			
gram per capita per day	48.7	48.5	
% of calories	8.6	9.6	10–15%
fruit^c^			
gram per capita per day	34.2	77.0	
vegetables^d^			>400 g
gram per capita per day	45.8	76.4	

^a^Food balance sheet information, 2005: FAOSTAT (FAO 2009*a*).

^b^Population nutrient intake goals defined by the WHO Expert Committee (WHO/FAO 2003).

^c^Fruit defined as: plantains, bananas, orange, lemons and limes, grapefruit and pomelos, tangerines, mandarins, clementines, satsumas, other citrus fruit, melons, watermelons, apples, apricots, avocados, cherries, figs, grapes, mangoes, papaya, peaches, pears, persimmons, pineapples, plums, quinces, blueberries, cranberries, gooseberries, raspberries, strawberries, kiwi, other fruits (fresh), dates, figs (dried), prunes, currants, raisins, other dried fruit.

^d^Vegetables defined as: beets, carrots, turnips, rutabagas/swedes, onions (green), onions (dry), artichokes, tomatoes, asparagus, cabbage, cauliflower, celery, kale, lettuce, spinach, beans (green), broad bean (green), chilli peppers, garlic, cucumbers, mushrooms, eggplant, peas (green), pumpkins, squash, gourds, okra, radishes and other vegetables.

Only a few studies have investigated the applicability of FBS food availability statistics for assessing dietary consumption in low-income country settings and generally conclude that FBS data may underestimate actual intake (Poleman 1981; Svedberg 1999), primarily because people grow, catch and process a large proportion of their diet that do not appear in country-level production statistics. For example, data on milk production and consumption in Bolivia, Kenya and Nepal indicate that only 13 per cent of milk is produced and traded in formal milk chains (Anderson *et al*. 2004). It is arguably of greater concern to have accurate measurement of food consumption in low- and middle-income countries where there remains under-nutrition coupled with the increasing transition to high-energy, low-nutrient diets. These transitions may not occur uniformly across a country or even within a household (FAO 2006), questioning the usefulness of country-level FBS for providing data that will inform nutrition policy. Nationally representative nutritional surveys are a more accurate and nuanced method of characterizing the diet of a population, and the widespread reliance on FBS food availability data in poorer countries has important implications for the limits of our understanding of diet in these settings, not least because of the paucity of FBS statistics from these regions.

## 5. Implications of the knowledge gap

The incomplete nature of the available agricultural production and dietary intake data poses significant limitations on our ability to provide guidance to policy makers on ensuring food security for all. Projected agricultural production estimates are based on global food availability data and the likely changes in availability in light of historical patterns (FAO [Bibr RSTB20100122C19][Bibr RSTB20100122C20]). Thus, the FAO estimates that by 2050 the global average daily calorie availability will reach 3050 kcal per person (FAO [Bibr RSTB20100122C19][Bibr RSTB20100122C20]). While this estimate suggests that there should be sufficiency in terms of calorie availability, it does not mean that in 2050 all nine billion inhabitants of the Earth will be able to consume a healthy diet.

Inaccuracies in measuring or estimating food consumption undermine our capacity to know whether we are currently able to feed the world healthily and to assess the impact of projected agricultural trends. It is noteworthy that in the millennium development goals (MDGs), the consumption-related indicator for reducing hunger (MDG 1C) is the proportion of the population below the minimum level of dietary energy consumption (based on food availability data), a statistic undermined by the limitations of FBS with its limited information on the distribution of food consumption, and also a statistic lacking any direct emphasis on dietary quality.

Influencing the future production and processing of food requires a thorough understanding of the impacts of a changing food system on health, which will in turn rely on accurate data from each stage of the food system: from production to consumption. A good understanding of what foodstuffs are being produced, imported and exported in different countries and regions not only allows surveillance of current production for nutritional planning, but provides a means of evaluating policy interventions aimed at improving production for nutritional (and other) goals or assessing other shocks to the food system, such as the recent global financial crisis.

As countries progress through economic and nutrition transitions, with a greater proportion of the diet becoming processed foods, the food system becomes increasingly complex, and traditional calculations of commodity availability are a poor proxy for consumption patterns of nutrients (Dowler & Ok Seo 1985). A thorough understanding of the impact of the changing food system on health will therefore require information on the combining, mixing and removing of nutrients during the manufacturing of processed products (FAO 2004) and/or detailed information on nutritional intakes. Although considered the ‘gold standard’ for monitoring population dietary intake, nationally representative data on food consumption are only available for a small minority of countries and this situation is unlikely to improve in the short term due to resource constraints.

## 6. Improving production and consumption estimates

We have shown that food availability data cannot be used interchangeably with food consumption data. Moreover, the accuracy of statistics behind food availability data is extremely variable, and it seems unlikely that current institutional incentives to improve the system will be adequate to significantly enhance data collection and analysis. Notwithstanding these concerns, accurate data on food consumption are a vital component of effective planning of public agricultural investments and for the implementation of sound public health nutrition policy. To improve our capacity to predict the health consequences of changes in agriculture and food systems, we propose the following areas for future work:

— improve FBS measurement, through more refined data collection and analysis to estimate food production;
— more extensive and representative individual dietary intake studies, focused on areas at risk of under-nutrition and those in dietary transition;
— better information on the mixing and processing of food, its nutritional content and the destination of processed foods; and
— enhanced data on waste at all stages of the food chain.

Such a list of data collection needs is, however, not new. For example, the Partnership in Statistics for Development in the twenty-first century (PARIS21) was established in 1999 to facilitate the collection of national statistics in low-income countries (www.paris21.org) but appears to have had little impact on the quality of statistics used to assess food availability in these settings. However, we suggest there are two recent trends that might shift this situation, one in the public and one in the private sector.

In recent years, the world has seen dramatic change and improvements in data collection for other aspects of the economies in low- and middle-income countries such as poverty data capture and analysis relating to the MDGs. There are strong arguments that, as the MDGs come to be reviewed towards 2015, there should also be a refinement in data collection and analysis processes to ensure that links between food production, processing and consumption can be placed in a systems framework that not only demonstrates access to food but also to the right balance of key nutrients. This will require substantial resources, but its linkage to globally agreed goals will make such investment more likely.

Secondly, the conditions are right today for public–private partnership approaches to healthier diets, with potential for greater collection of consumption data by the private sector. Major food manufacturers and retailers are increasingly aware of the significance of food quality, diet and health for social responsibility in relation to consumers, as indeed they are of the significance of agricultural production conditions for social and environmental responsibilities among suppliers. Moreover, through electronic data collection at the point of sale, major manufacturers and retailers are the repositories of at least some of the food production, processing, preference and purchase data for which there are public sector *lacunae*. While there remain considerable shortcomings in these data for assessing food consumption (no information on food distribution, waste and so on), they could represent an important untapped resource on patterns of food purchase in the retail sector. The dramatic spread of supermarkets in low- and middle-income countries (Reardon *et al*. 2003) may make such measurement particularly valuable there, where there is little public sector investment.

## 7. Looking ahead

The arguments presented so far address our need for a better understanding of the current relationship between agriculture and health. But they also apply to our desire to predict the health consequences of future agricultural change and to support the evaluation of different potential interventions to improve health through changing agriculture and food systems. Here we highlight a few trends and opportunities where improvements in measurement will be essential. Many of these relate to diet and nutrition, but others relate to factors resulting from the health ‘externalities’ of agricultural change.

With an increasingly clear picture of what constitutes a healthy diet, we will see a growing effort to ensure equity of access. The public sector will see this as a social obligation, and the private sector will be increasingly motivated to contribute, as is clear from the recent investment of food producers in research and promotion for healthy diets. We will be faced with a range of opportunities to improve diets, many of which exist today at some level, and include among others:

— breeding of more nutritious crop varieties, for instance, through breeding or engineering crop lines to express higher levels of vitamin A, iron and zinc (www.harvestplus.org), and breeding of animals, and/or modifying their diets, to produce healthier meat products (Stewart *et al*. 2001; Rymer *et al*. 2010);
— adding micro-nutrients to processed foods, as has been done in many countries with iodine (Zimmermann *et al*. 2008) and folic acid (Stevenson *et al*. 2000), and is increasingly a feature of the commercial development of processed foods;
— providing nutritional supplements, specifically to populations at particular risk of malnutrition, such as routine vitamin A supplementation during child immunization programmes (Goodman *et al*. 2000);
— campaigns to change consumer behaviour and encourage healthier diets, such as the UK ‘five a day’ programme to promote fruit and vegetable consumption (Department of Health 2010), or similar price-based initiatives from the food and food retail industry; and
— encouraging the production of healthier foods through targeted commodity production, import or export subsidies.

At the same time, there will be growing opportunity for a ‘do nothing’ strategy where negative nutrition-related health effects of dietary behaviours can be mitigated via medication such as statins to reduce LDL cholesterol (Delahoy *et al*. 2009).

These different agri-health interventions and others may have the potential to improve the health of all populations. Predicting their health outcomes will be essential to calculate the long-term health gains associated with the short-term private or public sector investment required, providing the basis for selecting—and selecting between—these different approaches for specific situations. For instance, the vitamin A-associated health benefits of uptake of new ‘golden rice’ varieties, genetically modified to express beta-carotene, has been calculated in terms of disability adjusted life years (Stein *et al*. 2006) which can in turn be used to calculate the rates of return on agricultural investment.

There will also be a need to measure non-dietary health effects of changes in agriculture and food systems, as exemplified by the ‘livestock revolution’, an increase in meat and dairy production to respond to growing demands of wealthier, urban populations in developing countries (Delgado *et al*. 1999). Much of the recent growth in the livestock protein supply has come from intensive monogastric systems and to some extent from a growth in milk production. The dramatic increases in livestock production have been celebrated, but this trend has also generated concerns about the contribution of meat and dairy products in the dietary transition and the growth of chronic diseases (Popkin 2009). Concerns have also been raised in relation to health externalities such as the impact on the livelihoods of traditional farmers (Haan *et al*. 2001; Hefferman 2002) and potential negative environment impacts (Steinfeld *et al*. 2006). There has been concern about growing problems with the control of transboundary animal diseases and more specifically the emergence and resurgence of dangerous zoonotic diseases (Greger 2007). There are pervasive arguments that recent rises in disease problems are related to changes in livestock production systems and the increase in livestock populations (Leibler *et al*. 2009), although the capacity to collect data and to analyse these systems continue to be weak.

These health externalities add to the challenge of developing agricultural systems that support health, but they also create indirect opportunities for health improvement. For example, a recent study estimates that reducing the production of animal source food products (especially but not only in high-income countries) could be an important strategy to achieve greenhouse gas mitigation targets. If reduced production also results in reduced consumption of animal source foods, it will represent an important health ‘co-benefit’ of an agri-environmental intervention (Friel *et al*. 2009).

## 8. Conclusions

The global agricultural system is primarily concerned with ensuring that sufficient food (in terms of calories) will be produced to feed the projected global population of nine billion in 2050 (FAO [Bibr RSTB20100122C19][Bibr RSTB20100122C20]). However, to tackle global public health problems associated with both under- and over-nutrition, healthy diets must be sufficient not just in calories but also in the balance of macronutrients, vitamins and minerals. Our increasingly sophisticated understanding of the association between diet and health should now prioritize health as a key driver of future agricultural production.

The quality and paucity of available information on food production and individual-level food consumption, especially in the most nutritionally challenged regions of the world, severely hampers our efforts to link agricultural production with health. Furthermore, limitations in the available evidence look set to increase as the food system becomes more complex and global in nature. It is clear that food availability statistics provide information that should not be used as an estimate of individual dietary consumption and that actual food consumption data will be needed to assess the impacts on health of future developments in the agricultural and food systems.

The enormous challenge of global food security is likely to stimulate considerable investment and innovation in agriculture and food science in the coming decades which will hopefully contribute to improving food supply at a global level. However, too narrow a focus on cereal improvements and calorie supply alone will not eradicate under-nutrition or address the health challenges arising from the nutrition transition. An integration of agricultural innovation and population health planning is required, based on matrices that will allow us to better understand the impact of the agriculture and food systems on population health.
